# Supplementary material for: Patterns of Intron Gain and Loss in Fungi
Source: PLoS Biol. 2004 Nov 30;2(12):e422. doi: 10.1371/journal.pbio.0020422 (PMC532390; doi:10.1371/journal.pbio.0020422)
Supplement: Table S1 — Also available at http://genes.mit.edu/NielsenEtAl/. (4.3 MB ZIP). [file pbio.0020422.st001.zip › NielsenEtAl/html/1190.html]

AN5014.1.NCU06661.1.MG04104.1.FG09974.1


```
 CLUSTAL W (1.82) Multiple Sequence Alignments - Introns Inserted


Sequence 1: NCU06661.1	126 aa
Sequence 2: MG04104.1	125 aa
Sequence 3: FG09974.1	124 aa
Sequence 4: AN5014.1	132 aa
Alignment Length: 134 aa
Number Identitical Residues: 73 aa
Alignment Score (without introns) 3366


MG04104.1 	MAPP~Q~T0KKT-GKSSKTTKK0FVINASQPVNDKIFDISAFEKFLNEKIKIDGRVGQLG
NCU06661.1	MAPI~T0K~KSAKGKGPKVTKK~FVINASQPASDKIFDVSAFEKFLTEKIKVEGRVGNLG
FG09974.1 	MAP-~Q~A0KKS-GKAQKQTKK0YIIDASQPASDKIFDVAAFEKFLQDRIKVEGRTNNLG
AN5014.1  	MAPA0V~A~RGR--KAQKVTQK~FIINASQPANDKIFDVSAFEKFLHDRIKVEGRVGNLG
          	***      :    *. * *:* ::*:****..*****::****** ::**::**..:**

MG04104.1 	DTIVISQVPDGKIEIVAHNELSGRYLKYL2--------TKKFLKKQQLRDWLRVVSTSKG
NCU06661.1	DAIKISQVGDGKIEIVAHNELSGRYLKYL2--------TKKFLKKMQLRDWLRVVSTSRG
FG09974.1 	DNVVVKQQGEGKIEIIAHNELSGRYLKYL2--------TKKFLKKQQLRDWLRVVSTSRG
AN5014.1  	DKVVISQVGDGKVEVVAHIPFSGRYLKYL2LTGAFVFRTKKYLKKQQLRDWLRVVSTSKG
          	* : :.*  :**:*::**  :********  :.:    ***:*** ************:*

MG04104.1 	VYELKFFNVVNDADDEDDE
NCU06661.1	VYELKFFNVVNDEAEEDEE
FG09974.1 	VYELKFFNVVNDEADEDDE
AN5014.1  	VYELRFYNVASEEADEDEE
          	****:*:**..:  :**:*
```
